# Revisiting the activate depress free and repeat (ADFR) paradigm: Clinical use of cyclic romosozumab

**DOI:** 10.1007/s11657-026-01750-5

**Published:** 2026-08-14

**Authors:** Kristyn Hare, Shawna Wheeler, Christina Morganti, Diane Krueger, Donald Kimmel, Neil Binkley

**Affiliations:** 1 Department of Orthopedics and Rehabilitation, University of Wisconsin-Madison, Madison, WI USA; 2 Osteoporosis Clinical Research Program, University of Wisconsin-Madison, 2870 University Ave, Suite 100, Madison, WI 53705 USA; 3 Department of Physiological Sciences, College of Veterinary Medicine, University of Florida, Gainesville, FL USA

**Keywords:** Osteoporosis; Treatment; Cyclic therapy, Romosozumab

## Abstract

***Summary*:**

Osteoporosis treatment does not normalize BMD. We hypothesized cyclic romosozumab dosing might provide greater BMD increases. Cyclic therapy (six romosozumab monthly doses then one denosumab dose) produced broadly similar BMD increases as previously reported for 12 romosozumab doses. A second cycle further increased spine and hip BMD but with a blunted response.

**Purpose:**

Evaluating novel osteoporosis medication approaches to maximize BMD is reasonable. A bone remodeling-based cyclic approach, i.e., activate, depress, free and repeat (ADFR), has been proposed. We hypothesized that short course romosozumab followed by denosumab represents an appropriate cycle. This exploratory retrospective case series reports six months of romosozumab treatment then one denosumab dose, followed by a second identical cycle.

**Methods:**

Women at very high fracture risk were offered cyclic therapy. DXA was obtained at baseline, 1 and 2 years. Data from 41 women treated for 1, and 18 for 2 years are reported. Percent BMD change from baseline was evaluated by ANOVA.

**Results:**

Patient mean (SD) age and BMI were 68.8 (6.6) years and 25.6 (5.0) kg/m^2^ respectively. Mean total hip T-score was -2.5, FRAX estimated hip fracture risk was 6.9%. After one cycle, mean ± SEM% BMD increase (all p < 0.001) from baseline at the lumbar spine (LS), total hip (TH) and femur neck (FN) was 12.6 ± 1.1%, 5.9 ± 0.8% and 4.4 ± 0.9% respectively. In 18 women completing a second cycle, BMD further increased to + 17.5% and + 8.7% at LS and TH, respectively. In a subset (*n* = 14) CTX was reduced from baseline (mean -78%) before cycle two with 11 below the lower limit of normal.

**Conclusion:**

At one year, six monthly romosozumab doses then one denosumab dose substantially increased BMD. A second cycle further increased LS and TH BMD, but the response was blunted perhaps reflecting persistent turnover suppression by denosumab. Evaluation of novel romosozumab dosing approaches is indicated.

**Supplementary information:**

The online version contains supplementary material available at 10.1007/s11657-026-01750-5.

## Introduction

Osteoporosis is a disease of low bone mass and deteriorated trabecular microarchitecture, resulting in increased fracture risk. Two broad classes of pharmacological agents, antiresorptive and anabolic, are used as monotherapy to reduce fracture risk. Continuous monotherapy with either class reduces vertebral fractures by ~ 80% and non-vertebral fractures by ~ 40% but does not usually normalize bone mineral density (BMD) [1, 2]. Combinations of two medications have been tested but provide, at best, only modest BMD advantages over continuous monotherapy [3–5]. Cyclic therapy with teriparatide, denosumab and raloxifene has been explored, again without substantial BMD advantage over continuous monotherapy [6–9]. Thus, it is currently not possible to “cure” osteoporosis.

Therefore, it is reasonable to consider additional approaches to increase BMD more than currently possible, ideally with approved agents. To this end, we considered two osteoporosis treatment paradigms initially proposed decades ago when no agents were specifically approved to reduce osteoporotic fracture risk [10, 11]. Both strategies exploit knowledge of adult skeletal remodeling and modeling systems and how those systems might be targeted by agents which affect bone cell function. Notably, Frost proposed a cyclic, bone physiology-based, paradigm called ADFR (Activate, Depress, Free, Repeat) [10, 12]. ADFR leverages the orderly, three phase sequence of bone cell activity; Activate, Resorb, Form in basic multicellular units (BMUs) of the adult remodeling system and was conceived to work entirely within that framework. ADFR was initially envisioned to utilize an agent that markedly increases activation frequency briefly, thereby activating a large cohort of synchronized BMUs. Subsequently, as the cohort of synchronized BMUs enters resorption phase five-seven days later, ADFR uses an antiresorptive to depress osteoclast activity during the two- to three-week resorption phase. Next, ADFR frees the cohort of BMUs from all treatment to finish the formation phase. Frost projected that all BMUs of the synchronized cohort would produce bone structural units (BSUs) with shallower than normal resorption cavities because resorption was inhibited specifically during their resorption phase. Frost also hoped that each BMU in the cohort would then form a normal amount of bone in the shallower resorption cavities during the formation phase. This would create a cohort of overfilled BSUs, thereby increasing BMD. Finally, but importantly, *“Repeating the sequence should add further increments”* [11]. As such, ADFR planned to use repeated ADF cycles, creating multiple cohorts of overfilled BSU’s, thereby increasing BMD into the normal range [10, 12]. Early studies of the ADFR paradigm, using oral phosphate or triiodothyronine followed by etidronate, among other agents, were undertaken with negative results [13, 14]. With the current availability of much more potent medications, revisiting an ADFR-like approach is reasonable.

Shortly after Frost envisioned ADFR, preclinical investigators proposed another bone physiology-based osteoporosis treatment paradigm; Activate, Restore, Maintain (ARM) to increase and subsequently preserve bone mass. This idea emerged from rat studies in which PGE2 increased modeling-based bone formation at and adjacent to quiescent bone surfaces and also increased endocortical remodeling-like activity [15–19]. During continued treatment, bone mass increases plateaued, however, upon PGE2 discontinuation, bone mass declined to pre-treatment levels. These investigators proposed the crucial maintain portion of ARM, taking advantage of the then newly discovered third generation bisphosphonates. This paradigm, i.e., the necessity to maintain BMD following cessation of anabolic therapy, is now widely recognized and implemented into clinical guidelines [20–22]. However, unlike Frost’s ADFR paradigm, the ARM approach proposed no cyclic therapy.

The approach of this exploratory descriptive retrospective case series combines the now widely accepted ARM approach with the repeat phase of Frost’s ADFR paradigm. We considered romosozumab as a potential agent to implement ADFR given its dual mode of action, i.e., formation stimulation and antiresorptive activity, and sought to optimize the romosozumab treatment duration and incorporate cyclic therapy. Review of multiple studies identified six months as a reasonable duration for romosozumab treatment as this duration increased lumbar spine and total hip BMD by ~ 10% and ~ 4%, respectively [1, 23–28]. Importantly, the anabolic effect of romosozumab largely dissipates by six months, providing independent support for courses shorter than 12 months [1, 29]. We hypothesized that six months of romosozumab alternating with a single dose of denosumab would constitute a reasonable cyclic approach. Here we report data from a retrospective case series of osteoporotic women treated with monthly romosozumab for six months, then one denosumab dose, then a second identical cycle begun at six months after the denosumab dose. As these data reflect clinical care, rather than a standard randomized prospective study, no control group exists.

## Methods

### Clinical cohort

All women reported here were evaluated by practitioners in a fracture liaison service/bone health optimization team (n = 4 providers; KH, SW, CM, NB). These patients were either seen after occurrence of a clinical fracture or through orthopedic surgeon referral for bone health optimization prior to elective orthopedic surgery. Clinical history, including consideration of potential secondary causes of bone loss and fracture history, was obtained at the initial evaluation. Routine laboratory assessment for secondary causes of bone loss and clinical DXA of the lumbar spine (LS), total hip (TH) and femur neck (FN) was obtained. After discussion of the rationale, women at very high fracture risk were offered cyclic therapy. Very high fracture risk determination was done by the individual practitioner based on the AACE guidelines, i.e., T-score < −3.0, high FRAX risk and recent fracture [20]. Many of these patients (20/41) had never received osteoporosis medications. Recommendations formally defining what constitutes “treatment naïve” patients have recently been published [30]. According to this definition [30], 20 of the remaining 21 patients were considered treatment naïve.

### Laboratory evaluation and DXA

Routine laboratory evaluation included serum chemistries, 25-hydroxyvitamin D (25[OH]D), parathyroid hormone (PTH) and phosphate. Additional laboratory assessment varied and was performed at the individual clinician’s discretion. All laboratory tests were performed in the clinical laboratory directed by the patient’s healthcare plan. When serum C-telopeptide (CTX) was obtained, at the ordering practitioner’s discretion, blood was drawn in the morning following an overnight fast.

Clinical DXA scans were obtained at the initial evaluation and after one and two years. Most scans (38/41 patients) were performed using GE Healthcare Prodigy or iDXA densitometers. All densitometers in our healthcare system are cross-calibrated GE Healthcare instruments. For those outside our system the same instrument was utilized for all scans. Three patients’ scans were obtained with Hologic instruments. All scans were obtained in routine clinical manner and were reviewed by the practitioner to validate accuracy. BMD of L1-4, lowest total hip, femur neck and one-third radius (when available) were obtained.

### Treatment approach

Cyclic therapy consisted of monthly romosozumab, 210 mg administered subcutaneously in clinic for six doses. One month after the sixth romosozumab injection, a single 60 mg denosumab subcutaneous injection was administered. A follow up clinic visit occurred approximately one year after the first romosozumab dose. At that time, a second identical romosozumab/denosumab cycle was initiated. In 18 patients completing this second cycle, a follow up clinic visit and DXA were performed at two years from treatment initiation.

### Data analysis

Data were compiled by EMR review performed by one practitioner (KH). Basic descriptive statistics were compiled. Percent BMD change from baseline to year 1 was evaluated by repeated measures ANOVA using JASP v 0.95.4 (University of Amsterdam; Amsterdam, NL). Percent BMD change from baseline to year 1 and from year 1 to year 2 was compared by paired T-test (*N* = 18).

## Results

### Patient cohort (Table 1)

All patients (*n* = 41) were postmenopausal White women who were at high fracture risk with a mean lowest T-score of −3.1. Prevalent vertebral and non-vertebral fractures were present in 39% and 68% with a FRAX major osteoporotic fracture risk of 23% and for hip fracture 6.9% (Table 1). Eighteen patients had completed the second romosozumab/denosumab cycle at the time of data collection for this report.

**Table 1:** Baseline demographic, DXA and laboratory data; *n* = 41 White women

Age (years)	68.8 ± 6.6	% with any prior fracture	90.2
BMI (kg/m^2^)	25.6 ± 5.0	If fracture, # of prior fractures	2.5 ± 1.6
L1-4 T-score	−2.1 ± 1.1	25(OH)D (ng/mL)	57.9 ± 22.2
TH T-score	−2.5 ± 0.7	PTH (pg/mL)	57.9 ± 22.5
FN T-score	−2.6 ± 0.5	Creatinine (mg/dL)	0.76 ± 0.15
0.3 radius T-score	−2.6 ± 1.0	PO4 (mg/dL)	3.5 ± 0.38
Lowest T-score	−3.1 ± 0.70	Albumin (g/dL)	4.0 ± 0.32
MOF (%)	23.2 ± 7.2	Calcium (mg/dL)	9.6 ± 0.35
Hip (%)	6.9 ± 4.1	CTX (pg/mL)	566 ± 248
% with prior vertebral fracture	39.0		
% with prior non vertebral fracture	68.3		

Data as mean ± SD

### Treatment

All prescribed romosozumab and denosumab doses in all patients were administered as scheduled. In the first cycle, a denosumab dose was administered at a mean of 6.8 months after the first romosozumab dose. No adverse events leading to treatment discontinuation were observed. No major adverse cardiac events occurred. All patients were routinely instructed to receive 1,000 −1,200 mg calcium daily. Daily vitamin D_3_ supplementation was recommended if circulating 25(OH)D was < 30 ng/mL.

### BMD

**Fig. 1:**
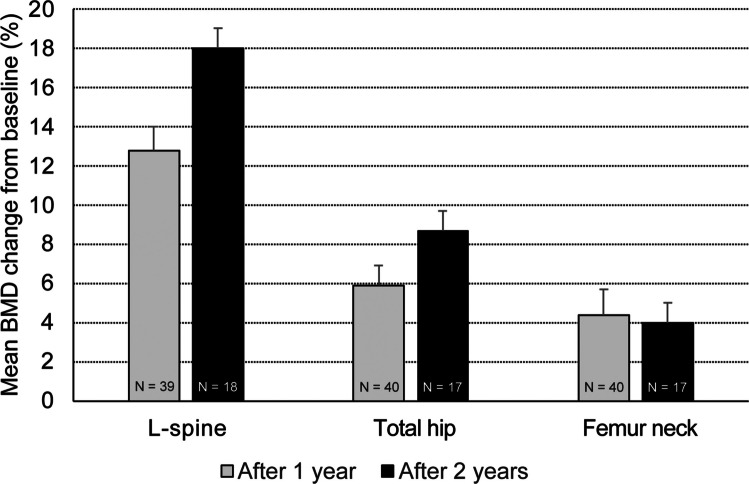
Mean percent BMD change from baseline. One cycle of romosozumab/denosumab increased BMD (*p* < 0.001) at the lumbar spine, total hip and femur neck at one year. In a subset (*n* = 18) of patients who received a second romosozumab/denosumab cycle, percent BMD further increased (*p* < 0.001) at the lumbar spine and total hip from year 1 to 2. The mean ± SEM percent BMD change from year 1 to 2 (using year 1 as a new “baseline”) was + 4.7 ± 0.6% at the lumbar spine (*p* < 0.001) and + 2.1 ± 0.6% at the total hip (*p* = 0.001), while BMD change at the femur neck, −0.3 ± 1.3% (*p* = 0.80) was not significant. The BMD change from year 1 to year 2 was significantly less than in year 1. Data presented as mean (± SEM) percent change from baseline

After one year, the mean ± SEM percent BMD change from baseline (all *p* < 0.0001) at the LS, TH and FN was + 12.6 ± 1.1%, + 5.9 ± 0.8%, and + 4.4 ± 0.9%, respectively (Fig. 1). In the 18 patients completing two years of cyclic therapy, the mean ± SEM percent BMD change from baseline (all *p* < 0.001) at the LS, TH, and FN was + 17.5 ± 1.3%, + 8.7 ± 1.2%, and + 4.0 ± 0.8% (Fig. 1). In these 18 patients, the mean ± SEM percent BMD change from year 1 to 2 (using year 1 as a new “baseline”) was + 4.7 ± 0.6% at the LS (*p* < 0.001) and + 2.1 ± 0.6% at the TH (*p* = 0.001). BMD at the FN did not change from year 1 to 2; −0.3 ± 1.3% (*p* = 0.80). Though the LS and TH percent BMD change from year 1 to year 2 was significant, the LS and TH BMD increase from year 1 to year 2 was significantly less (*p* < 0.001) than from baseline to year 1.

### CTX

**Fig. 2:**
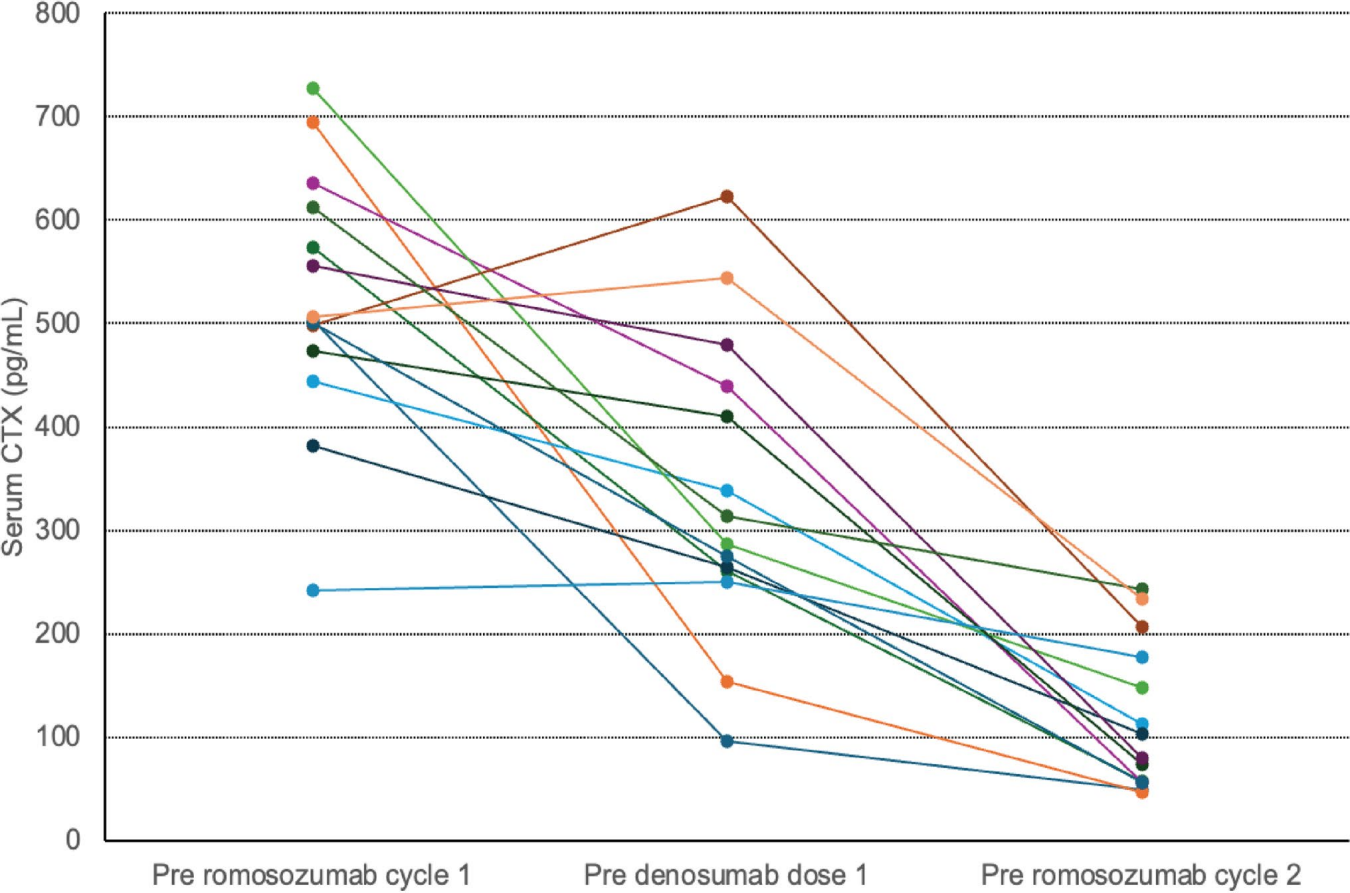
Individual patient CTX data. In the 14 women with CTX obtained at three timepoints; prior to cycle 1, just before denosumab, and again just prior to initiation of romosozumab cycle 2, the value was substantially reduced (*p* < 0.001) from baseline with 11/14 being ≤ 177 pg/mL (the lower limit of postmenopausal normal range)

In a subset (*n* = 14) with data available from baseline, six months, and one year, serum CTX was reduced after six romosozumab injections from 525 ± 126 to 338 ± 146 pg/mL (*p* < 0.001). Subsequently, at 1 year, just prior to initiation of the second romosozumab cycle, CTX was further reduced (*p* < 0.001) to 117 ± 71 pg/mL yielding a total mean reduction of ~ 78% from baseline. Prior to initiating the second cycle, 11/14 patient CTX values were ≤ 177 pg/mL, the lower limit of normal for postmenopausal women (Fig. 2).

## Discussion

Current optimal osteoporosis therapy for those at very high fracture risk consists of initial anabolic treatment followed by antiresorptive medication [31]. This approach generally increases BMD, ultimately by less than 20%, does not return BMD to normal, and does not prevent all fractures. As such, consideration of alternative treatment strategies is reasonable. In this clinical cohort, six monthly doses of romosozumab followed by a single denosumab dose substantially increased BMD. Indeed, the BMD increases observed here with one cycle are broadly similar to that observed with 12 months of continuous romosozumab [1].

In a subset of these patients, a second romosozumab/denosumab cycle further increased lumbar spine and total hip BMD. However, with the second cycle, the BMD increases were less marked perhaps reflecting persistent remodeling suppression by denosumab which would could theoretically inhibit remodeling-associated BMD gain caused by romosozumab. These results are broadly similar to reports demonstrating comparable BMD gains with less than one year of romosozumab [24–28], notably as little as three monthly doses [24, 32 ]. These data support continued evaluation of romosozumab courses shorter than one year, followed by an antiresorptive.

Acknowledging that direct comparison of these data with prior romosozumab studies is challenging, it is nonetheless reasonable to explore how these BMD increases relate to previously published data. In this regard, the BMD increases reported here are remarkably similar to the FRAME study in which 12 months of romosozumab was followed by two doses of denosumab (Supplemental Fig. 1) [1]. Such generally similar BMD increments are encouraging, as real-world patients differ from clinical trial participants, potentially in multiple ways which often include more comorbidities that would have led to exclusion from Phase III trials [33]. Indeed, the clinical patients in this cohort had numerically higher vertebral and non-vertebral fracture prevalence (39% and 69%, respectively) than participants in the FRAME study (19% and 22%, respectively) [1]. One might hypothesize that these real-world patients, likely with more comorbities and higher prevalent fracture rate, might have a less robust response to romosozumab. As such, the generally similar BMD increases seen here support investigation of novel dosing approaches.

Achieving further significant increases in lumbar spine and total hip BMD with a second cycle is encouraging. This is especially true when one considers the available CTX data imply that bone remodeling was still suppressed at the start of the second cycle. This persistence could be expected to block activation of new BMUs, thus preventing any BMD increase associated with remodeling activity. Thus, the second cycle BMD increase may be attributed to modeling-induced BMD gain induced by romosozumab which requires no prior bone resorption [34]. This leads to consideration of customizing second cycle start time based on the CTX value, in hopes of achieving even larger second cycle BMD gains.

Achieving less BMD gain with the second cycle emphasizes the widely recognized importance of treatment timing [3, 31, 35]. Specifically, prior use of bisphosphonates or denosumab blunts the BMD increase attained with anabolic agents [36–38]. Appreciation of such blunting led to our use of denosumab, as opposed to a bisphosphonate, in this cyclic regimen due to its shorter-term suppression of bone resorption and lack of skeletal persistence. Unfortunately, in patients where CTX was measured just prior to initiating the second romosozumab cycle, persistent bone remodeling suppression was observed. It seems plausible that this played a role in the less robust BMD increase observed. This indicates that our approach did not adequately adhere to Frost’s paradigm in that a period “free” from anti-resorptive activity was not achieved with our timeframe. Perhaps delaying initiation of the second treatment cycle until CTX has risen substantially might result in greater BMD gains. Alternatively, use of a less potent antiresorptive, such as raloxifene, could be considered. Finally, given that romosozumab has inherent antiresorptive activity, simply cycling this agent could also be considered, however the prompt increase in CTX seen when romosozumab is followed by placebo argues against such an approach [39].

It is appropriate to consider whether the romosozumab dosing duration used here is optimal for cyclic treatment. Based upon previously reported P1NP data, the romosozumab anabolic effect is maximal at one month and rapidly declines to near baseline by three to six months of continuous monthly treatment [1, 29, 38]. The mechanism(s) underlying this rapid decline in bone anabolic efficacy is inadequately understood. It has been suggested that this loss of anabolic response is associated with increased expression of Wnt antagonists [40]. It is indeed plausible that anabolic attenuation reflects compensatory increases in bone formation inhibitors such as Dkk1 or alternatively exhaustion of the mesenchymal stem cell pool [41, 42]. If osteoblast progenitor depletion is operative, perhaps BMD gains that have been reported previously, and achieved in this patient cohort, have reached maximums achievable with simple cyclic therapy. If so, approaches that may expand the osteogenic stem cell population prior to or even during bone anabolic therapy might be of major therapeutic benefit [43, 44]. Regardless of the underlying mechanism(s), perhaps even shorter courses of romosozumab during a cycle, such as one to three consecutive monthly injections [32] could perform as well as the six monthly injections used here. Clinical studies evaluating cycles with shorter romosozumab courses are indicated.

As noted previously, the paradigm to increase bone mass by sequential manipulation of osteoclasts and osteoblasts is far from a new concept, being proposed almost 50 years ago [10]. Early studies of this approach were unsuccessful, quite likely reflecting the lack of adequate therapeutic agents [10]. Thus, moving Frost’s somewhat more complex paradigm forward has awaited improved anabolic and antiresorptive agents which have become available over years of clinical trials. Perhaps prophetically, Frost suggested that *“X, the unknown which remains yet undiscovered”* could serve as an activator and depressor, as is now seen with romosozumab. Further, and optimistically, Frost stated *“The sequence is repeated as often as needed to add further increments”* and upon revisiting the concept in 1984, he recognized that operationalizing this approach *“would require knowledge of the sequences, timings and durations”* of various agents [12]. In this regard, perhaps improved knowledge regarding therapy duration could avoid the denosumab “hangover,” referred to above. Improved understanding of antiresorptive persistence duration is needed.

As this is a report of clinical care, not a randomized prospective study, multiple limitations are worthy of note. Notably, no control group existed to allow direct comparison of cyclic therapy with 12 months continuous romosozumab followed by antiresorptive treatment. Moreover, in a small study of short duration any treatment effect on fractures could not be evaluated. However, with the recent FDA approval of the FNIH-ASBMR SABRE initiative qualifying total hip BMD change as a surrogate for fracture reduction efficacy, this limitation becomes less important. Indeed, the total hip percent BMD increase observed here substantially exceeds the SABRE surrogate threshold effect to reduce vertebral, hip and non-vertebral fractures of ~ 1.4% to 3.2% [45, 46]. Additionally, while all DXA images and data were reviewed by the providers, not all scans were performed in our healthcare system in which all scanners are cross calibrated, thus various densitometers were used which might influence BMD change. Use of percentage change over time, rather than absolute BMD was utilized in attempt to mitigate this weakness. While routine laboratory evaluation was performed, individual providers had discretion regarding additional laboratory testing and as such not all patients had CTX measurement prior to cycle two. Other limitations include being performed in a single healthcare system, a White patient cohort and small cohort size. Clearly, larger randomized studies are needed to draw robust conclusions. Finally, a single sequence of romosozumab treatment and a single antiresorptive (denosumab) were utilized. Multiple other approaches are possible.

In conclusion, in this exploratory, hypothesis-generating clinical patient series, two cycles of romosozumab/denosumab substantially increased lumbar spine and total hip BMD, with the second cycle providing significantly less increment than the first. Six months of romosozumab followed by one denosumab dose substantially increased BMD, similar to that previously reported for 12 months of monthly romosozumab. The less vigorous BMD gain during the second cycle perhaps reflects persistent remodeling suppression by denosumab at the time of initiating the second cycle. These exploratory observations support further evaluation in larger, randomized controlled trials of novel dosing approaches, perhaps using shorter romosozumab courses, different antiresorptive agent(s) and potentially a longer customized period to partially or fully “free” the skeleton from antiresorptive activity.

## Supplementary information

Below is the link to the electronic supplementary material.

ESM 1 Supplemental Fig. 1. Mean percent BMD change over two years; cyclic romosozumab/denosumab vs. FRAME. To explore BMD change observed in this clinical cohort with what might be expected with one year of romosozumab followed by one year of densosumab, i.e., the approach utilized in the romosozumab FRAME study, mean lumbar spine, total hip and femur neck BMD percent changes over two years are presented here. Formal statistical comparison was not attempted, but numerically similar BMD changes were observed. Cyclic data as mean percent change from baseline (SEM); FRAME as mean percent change from baseline (PNG 236 KB)

High resolution image (TIF 11.7 MB)
